# Determine knowledge and belief of Somalian young women about breast cancer and breast self-examination with champion health belief model: a cross-sectional study

**DOI:** 10.1186/s12911-022-02065-4

**Published:** 2022-12-08

**Authors:** Şeyma Zehra Altunkurek, Samira Hassan Mohamed

**Affiliations:** 1 Public Health Nursing Department, University of Health Sciences Gulhane Faculty of Nursing, Ankara, Turkey; 2 Department of Nursing, University of Health Sciences, Mogadishu, Somalia

**Keywords:** Breast cancer, Breast self-examination, Champion’s Health Belief Model Scale, Knowledge, Somalian women

## Abstract

**Background:**

Breast cancer (BC) is an important reason for mortality rates in Somalian women. In Somalia, many women are late in applying to the hospital for the diagnosis of BC. Breast self-examination (BSE) is considered an important early detection method for BC in encouraging women to learn to practice BSE, especially for women in developing countries. This study purposed to determine knowledge, and belief of BC and BSE and BSE practice among women in Mogadishu, Somalia using the champion health belief model (CHBM).

**Methods:**

This cross-sectional study was conducted on 413 women who were between 18 and 49 years of age. The data were collected by using sociodemographic variables (age, marital status, education level, income status), Champion’s Health Belief Model Scale (CHBMS), and an introductory questionnaire with questions about BC and BSE and between October 2020 and January 2021 in Mogadishu, Somalia. Further descriptive statistics, the Mann–Whitney U test, and Kruskal–Wallis analysis test were used to assess data that were not normally distributed.

**Results:**

Average age of participants was 22 ± 11.21 years. Only 35.4% of participants had information about BC, 37.8% had heard about BSE before, 25.2% knew BSE, and only 17.2% had done it. Income status, marital status, and age of first birth family were significantly associated with perceived sensitivity, health motivation, convenience, perceived benefits, and self-efficacy for BSE. Overall, the total scores of CHBMS were significantly higher among those who had heard and knowledge about BSE and practiced clinical breast examination (CBE). For the sub-dimensions of perceived sensitivity, health motivation, perceived benefits, barrier, and self-efficacy BSE with hearing about BSE, practicing BSE, knowing to practice BSE, knowing early detection methods of BC and practice CBE significant differences were observed (*P* < 0.005).

**Conclusion:**

This study showed that BSE practice among Somalian women was very low, and they don’t have sufficient knowledge about BSE and BC. Furthermore, this study revealed that many CHBMS significantly related to BSE practice in Somalian women, suggesting that BSE health education programs with CHBMS.

## Background

Breast cancer (BC) ranks first in the world as an important health concern in terms of morbidity and mortality rates among women [1]. The incidence of BC accounts for one in four cancer cases and one in six cancer deaths worldwide. BC ranks first among the five important cancer types in women worldwide according to data from the International Agency for Research on Cancer [2]. Moreover, 2.3 million new BC cases and 685,000 deaths were recorded in 2020 [2].

In the last two decades, breast cancer has become a serious public health problem in developing countries, with the highest incidence of cancer and related deaths among women [3]. In developing countries, the burden of cancer is increasing due to population growth as well as risk factors associated with an economic transition such as smoking, obesity, physical inactivity, and reproductive behaviors [4]. According to Globocan 2020 data, approximately 1.9 thousand women were diagnosed with BC in Somalia, one of the developing countries. In respect of these data, BC is an important genus of cancer in Somalia and ranks first among cancer-related deaths [5].

Early diagnosis is very important to improve the BC treatment process, outcomes and survival. Early diaglnosis and treatment are effective in reducing BC-related death rates [6]. Mammography, BSE, CBE, and breast ultrasonography are recommended for the early diagnosis of BC [7–9]. Mammography is an effective method of choice for the early detection of breast cancer. However, it is not used much in developing countries due to its high cost and limited availability. Therefore, BSE is less reliable but is becoming a convenient and cost-effective method in developing countries [10]. However, women in developing countries do not perform breast self-examination for various reasons [11].

BSE is an examination performed by every woman after the age of 20. It is economical, easy to apply, safe, non-invasive, does not require a special material or tool; and it is an effective breast cancer screening method that takes only five minutes to apply. [12]. However, there is controversy about its effectiveness of routine BSE in the early detection of BC [13, 14]. In 2009, the US Preventive Services Task Force concluded that there was insufficient evidence that teaching or practicing routine BSE is beneficial or not helpful [15]. On the other hand, it was reported that BSE can help in reducing BC mortality rates. BSE is recommended in screening programs as it is thought to increase women’s knowledge of the structure of normal breast tissue and increase women’s awareness of this issue [16, 17]. Considering the relative benefits of BSE, its implementation remains low. There are hardly any studies on the subject in the African region. Studies show that BSE practices are very low [18].

Studies in Middle East countries showed that BSE practice between Saudi, Iranian and Turkish women was 41.6, 41.9 and 39.5% respectively [19, 20]. In Qatar, 24.9% of women know BSE as an arly diagnosis method and only 18.7% of women practiced BSE [21]. The study on female secondary school teachers in Nigeria indicated that most of the participants 95.6% had heard about BSE and 54.8% of them had done BSE before [22]. On the contrary, in developed countries such as the United Kingdom (UK) and Greek studies showed that women performed BSE %50, %71.3 respectively. Moreover, in Greek, 91.5% of participants knew about BSE [23, 24].

Breast early diagnosis practices are affected by severity, benefit, and barrier perceptions via a reasoning process that includes personal and community impacts and attitudes [25]. Cognitive-behavioral models have been developed to regulate and adopt preventive health behaviors for BSE application and BC screening. The Health Belief Model (HBM) is the first most and widely used [26]. The core elements of HBM focus on individual beliefs about health conditions and are predictive of individual health-related behaviors. The model identifies critical factors influencing health behaviors as an individual’s perceived illness or threat of illness (perceived susceptibility), belief in the severity of consequences (perceived severity), perceived benefits of action, perceived barriers to action, and exposure to motivators. action (action cues) and confidence in one’s ability to succeed (self-efficacy) [27]. Lately, researchers have noticed that women’s breast cancer early diagnosis practices follow their health beliefs. This theoretical framework explains the relationship between the beliefs and behaviors of the individual, as well as what is effective in the formation of the individual’s health-related behaviors and what motivates them to take action [29]. According to the HBM, women who are sensitive to BC, perceive BC as a serious disease, ha have a low perception of disability, and high benefits perception will apply BSE more [8].

Published data from African women show that BC has increased recently [30]. Furthermore, the increased incidence of BC in developing countries, coupled with insufficient resources for early detection and treatment programs and consequent disproportionately high death rates, has made breast cancer a major cause of premature death in these underdeveloped countries [31]. On the other hand, there is no information the concerning health perception of Somalian women, the sample of their health beliefs, and their relationship with their BC and BSE knowledge, and practice. Thus, the aim of this study was conducted to define the role of the health belief model in the practice of early diagnosis of BC and the knowledge, about BC and BSE among Somalian women.

## Method

The study is noticed using the STROBE Statement.

## Study setting and samples

This study is a descriptive cross-sectional design to determine the knowledge and beliefs of young women about BC and BSE. Participants were recruited from the hospital’s outpatient clinic and the university of Vocational school, in Mogadishu Somalia. The study was conducted between October 2020 and January 2021.

## Sample

The sample size indicated for this research was defined by using the formula to forecast a single population proportion, supposing that 22.1% of women have ever practiced BSE [32]. 95% CI, 5% margin of error, at alpha 0.05.

1 $$n = \frac{{\left( {z\frac{a}{2}} \right)^{2} *p\left( {1 - P} \right)}}{{d^{2} }}\quad n = \frac{{\left( {1.96} \right)^{2} *0.221\left( {0.797} \right)}}{{0.05^{2} }} = 270$$

The formula set is n = 270. After adding 5% for non-response, and multiplying by 1.5 for study design effect, and as a result sample size was 413 women. The exclusion criteria were the history of malignant tumors on the breast.

## Questionnaires forms

We constructed a demographic information form, the information form for BC and BSE, and the Champion’s Health Belief Model Scale (CHBMS). Demographic information form sought such as age, working, education, marital and income status, etc. Questions to ascertain knowledge about BC, BSE, and early diagnosis methods, hearing about BC, and having BC in relatives. The questionnaires were improved by the researchers after a vast literature review. Content validity has been approved by experts. The questionnaire was pretested with 20 participants that were not part of the research. After pretesting some of the questions were put differently for rather precision while others were taken out.

## Champion’s Health Belief Model Scale (CHBMS)

This scale has been improved by Champion in 1984. This form was revised finally in 1999 for the health beliefs about BC and BSE [27]. We used the Turkish version of the CHBMS (the T-CHBMS) to examine the HBM [26]. The CHBMS is specially purposed at about BC and BSE and consists of 42 items with 5-point Likert scales ranging from ‘strongly disagree’ [1] to ‘strongly agree’ [5]. The scale includes 42 Likert-type items in six sub-dimensions which are perceived severity, perceived sensitivity and benefits of BSE, BSE barriers, self-efficacy, and health motivation. The scale ranges from 1 to 5-point: 1, strongly disagree; 5, strongly agree. The highest scores on each subscale are 3–15 for perceived sensitivity, 7–35 for perceived severity, 4–20 for benefits of BSE, 11–55 for BSE barriers, 10–50 for self-efficacy, and 7–35 for health motivation. Higher scores demonstrate more positive views and attitudes towards health for all subdimensions, except the BSE barriers subscale, where higher scores show more disabilities [26]. Confirmatory factor analysis was used to evaluate the construct validity of the scale in this study, and the total Cronbach’s alpha value of the scale was found to be 0.759, and the alpha coefficient for subscales was between 0.713 and 0.769. In this study, the item-total correlation ranged from r = 0.35 to r = 0.70.

### Data analysis

The Statistical Package for Social Sciences (IBM SPSS Statistics 19; SPSS Inc, Chicago, Illinois) was used for analysis. Demographic data and data related to the study variables were analyzed by using descriptive statistics. Mann–Whitney U test and Kruskal–Wallis analysis test was used to assess data that were not normally distributed. *p* < 0.05 was considered statistically significant.

## Results

The mean age of the participants was 22 ± 11.21 years, and it was determined as min18-max 49. It was reported that 69.5% did not work of the women who participated in the study 72.2% had a university education level, and 58.1% had a medium economic status, 61.5% were single. About 35.6% of the participants have children, 45.6% of the women have 6–10 children, and 67.3% and of them have a first birth between 15 and 20 years (Table 1).

**Table 1:** Socio-demographic characteristics of the young women (n = 413)

Sociodemographic Characteristics	n	%
Age		
18–28	282	68.3
29–38	53	12.8
39–49	78	18.9
Working status		
Yes	126	30.5
No	287	69.5
Education status		
Primary school	35	8.5
Secondary school	29	7.0
High school	51	12.3
University and above	298	72.2
Income status		
Less than income	136	32.9
Income is equal to expenses	240	58.1
More than income	37	9.0
Marital status		
Married	159	38.5
Single	254	61.5
Do you have children		
Yes	147	35.6
No	266	64.4
Number of children (n = 147)		
1–5	66	44.9
6–10	67	45.6
11–15	14	9.5
Age at first birth (n = 147)		
15–20	99	67.3
21–25	42	28.6
26–30	6	4.1

About 64.6% of the participants and hdidknownowBC, 62.62% had never heard of BSE before and 74had no knowledge about BSE, 82.8% had not done BSE before, and 84% did not know how BSE was. It was determined that 91.5% of the participants did not have breast ultrasoundultrasounds1.8% did not have information about BC early diagnosis methods, and 94.2% did not have a CBE (Table 2).

**Table 2:** Young Women’s Knowledge breast cancer, BSE and practices on BSE (n = 413)

Variable	N	%
Knowledge of BC		
Yes	146	35.4
No	267	64.6
Family history of BC		
Yes	1	0.2
No	412	99.8
Have you heard about BSE		
Yes	156	37.8
No	257	62.2
Knowledge of BSE		
Yes	104	25.2
No	309	74.8
Have you done BSE before		
Yes	71	17.2
No	342	82.8
To know how to do BSE		
Yes	66	16.0
No	347	84.0
Have you had breast ultrasonography before		
Yes	35	8.5
No	378	91.5
Knowledge about early detection methods of BC		
Yes	75	18.2
No	338	81.8
Have you ever had a clinical breast exam		
Yes	24	5.8
No	389	94.2

*BC* breast cancer, *BSE* breast self examination

The participants’ mean CHBMS score was 109.37 ± 26.68; the minimum score obtained was 42, and the maximum score obtained was 171. When the participants’ CHBMS sub-dimension scores were evaluated, the lowest sensitivity was 5.71 ± 2.66 and the highest was 25.77 ± 8.37 BSE barriers subdimensions (Table 3).

**Table 3:** Young women’s CHBMS scores (n = 413)

Sub-dimensions	Possible score range	Min-Max	Mean ± SD
Sensitivity	3–15	3–15	5.71 ± 2.66
Severity	7–35	7–35	18.76 ± 6.47
HeaMotivationtion	7–35	6–35	21.77 ± 7.92
BSE benefits	4–20	4–20	12.74 ± 4.66
BSE barriers	11–55	11–49	25.77 ± 8.37
BSE self-efficacy	10–50	10–50	24.61 ± 8.96
Total CHBMS	42–210	42–171	109.37 ± 26.68

*CHBMS* Champion’s Health Belief Model Scale, *BSE* breast self examination

In the study, the results of the comparison of the CHBMS total score and sub-dimension mean scores of women with some sociodemographic characteristics are presented in Table 4. When the economic situation of the women participating in the research is evaluated; the sensitivity sub-dimension mean score of those with good economic status was determined as 6.21 ± 3.24, and a statistically significant difference was found (*p* = 0.01). When the economic situation and the health motivation sub-dimension were compared, it was statistically significant (*p* = 0.02). When the marriage status of the participants is examined; the BSE benefits sub-dimension mean scores of those who were married were higher (13.40 ± 4.46), and a statistically significant difference was found (*p* = 0.03) (Table 4).

**Table 4:** Distribution of mean scores of CHBMS and Subscales by sociodemographic characteristics (n = 413)

Demographic characteristics	CHBMS total scores		Sensitivity	Severity	HealtMotivationon	BSE benefits	BSE barriers	BSE self-efficacy
	n (n = 413)	X ± SD	X ± SD	X ± SD	X ± SD	X ± SD	X ± SD	X ± SD
Age								
18–28	282	109.16 ± 28.02	5.69 ± 2.64	18.81 ± 6.52	21.38 ± 7.99	12.51 ± 4.75	25.83 ± 8.71	24.92 ± 9.35
29–38	53	109.25 ± 22.65	5.28 ± 2.55	18.60 ± 6.19	22.98 ± 7.70	13.83 ± 4.27	24.28 ± 6.99	24.26 ± 8.46
39–49	78	110.22 ± 24.73	6.08 ± 2.76	18.70 ± 6.58	22.34 ± 7.7	12.84 ± 4.48	26.55 ± 7.92	23.67 ± 7.88
Statistics	χ^2^ = 0.736 *P* = 0.69		χ^2^ = 3.73 *P* = 0.15	χ^2^ = 0.86 *P* = 0.82	χ^2^ = 2.39 *P* = 0.30	χ^2^ = 3.02 *P* = 0.22	χ^2^ = 3.82 *P* = 0.14	χ^2^ = 1.63 *P* = 0.44
Working status								
Yes	126	110.17 ± 25.67	5.53 ± 2.50	18.44 ± 6.08	22.02 ± 8.27	12.95 ± 4.55	25.92 ± 8.71	25.28 ± 9.09
No	287	109.02 ± 27.23	5.80 ± 2.72	18.90 ± 6.64	21.657.76	12.65 ± 4.70	26.70 ± 8.22	24.30 ± 8.92
Statistics	Z = 0.05 *P* = 0.95		Z = 0.86 *P* = 0.39	Z = 0.75 *P* = 0.56	Z = 0.57 *P* = 0.56	Z = 0.73 *P* = 0.70	Z = 0.02 *P* = 0.97	Z = 1.00 *P* = 0.31
Education status								
Primary school	35	103.06 ± 32.56	6.17 ± 3.13	16.45 ± 6.90	20.25 ± 8.16	2.65 ± 4.75	25.17 ± 9.04	22.34 ± 9.35
Secondary school	29	113.83 ± 29.54	7.03 ± 2.51	20.62 ± 6.07	21.00 ± 7.77	12.86 ± 4.16	27.27 ± 9.07	25.03 ± 8.78
High school	51	109.63 ± 25.	5.64 ± 2.59	18.70 ± 6.83	21.54 ± 8.10	12.15 ± 5.10	26.66 ± 7.87	24.90 ± 7.20
University and above	298	109.64 ± 25.88	5.55 ± 2.59	18.86 ± 6.35	22.06 ± 7.88	12.84 ± 4.63	25.54 ± 8.31	24.77 ± 9.22
Statistics	χ^2^ = 3.24 *P* = 0.35		χ^2^ = 10.21 *P* = 0.17	χ^2^ = 7.17 *P* = 0.06	χ^2^ = 1.95 *P* = 0.58	χ^2^ = 0.92 *P* = 0.81	χ^2^ = 2.23 *P* = 0.52	χ^2^ = 2.38 *P* = 0.49
Income status								
Less than income	136	110.89 ± 25.65	6.12 ± 2.65	19.42 ± 5.70	21.21 ± 7.36	12.88 ± 4.20	26.05 ± 8.19	25.18 ± 8.40
Income is equal to expenses	240	109.13 ± 27.34	5.4 ± 2.53	18.59 ± 6.92	22.51 ± 8.10	12.79 ± 4.96	25.45 ± 8.48	24.36 ± 8.56
More than income	37	105.41 ± 26.95	6.21 ± 3.24	17.45 ± 5.98	19.00 ± 8.07	11.86 ± 4.24	26.83 ± 8.38	24.02 ± 10.06
Statistics	χ^2^ = 0.99 *P* = 0.60		χ^2^ = 8.15 *P* = 0.01*	χ^2^ = 3.02 *P* = 0.22	χ^2^ = 8.92 *P* = 0.02*	χ^2^ = 2.58 *P* = 0.27	χ^2^ = 1.77 *P* = 0.41	χ^2^ = 0.88 *P* = 0.64
Marital status								
Married	159	110.35 ± 25.69	5.61 ± 2.59	18.84 ± 6.59	22.21 ± 8.04	13.40 ± 4.46	26.09 ± 7.35	24.16 ± 8.76
Single	254	108.76 ± 27.40	5.78 ± 2.70	18.71 ± 6.41	21.49 ± 7.83	12.33 ± 4.74	25.57 ± 8.64	24.87 ± 9.11
Statistics	Z = 0.20 *P* = 0.83		Z = 0.60 *P* = 0.54	Z = 0.36 *P* = 0.97	Z = 0.98 *P* = 0.32	Z = 2.06 *P* = 0.03*	Z = 0.63 *P* = 0.52	Z = 0.79 *P* = 0.42
Do you have children?								
Yes	147	109.90 ± 25.94	5.69 ± 2.63	18.78 ± 6.67	21.91 ± 8.11	13.26 ± 4.48	26.12 ± 7.92	24.10 ± 8.95
No	266	109.08 ± 27.21	55.73 ± 2.68	18.76 ± 6.37	21.68 ± 7.81	12.45 ± 4.73	25.57 ± 8.61	24.87 ± 8.99
Statistics	Z = 0.09 *P* = 0.92		Z = 0.14 *P* = 0.88*	Z = 0.10 *P* = 0.91	Z = 0.40 *P* = 0.68	Z = 1.44 *P* = 0.14	Z = 0.84 *P* = 0.39	Z = 0.78 *P* = 0.43
Number of children (n = 147)								
1–5	66	110.47 ± 26.88	5.60 ± 2.65	18.53 ± 6.65	20.80 ± 8.28	13.59 ± 4.52	27.15 ± 8.29	24.78 ± 9.53
6–10	67	109.85 ± 22.00	5.56 ± 2.56	19.51 ± 6.17	22.74 ± 7.81	13.12 ± 4.13	25.60 ± 7.43	23.30 ± 8.39
11–15	14	109.79 ± 37.97	6.92 ± 2.75	17.42 ± 8.57	22.85 ± 8.82	12.78 ± 5.91	24.21 ± 8.39	25.57 ± 8.61
Statistics	χ^2^ = 0.52 *P* = 0.76		χ^2^ = 3.45 *P* = 0.17	χ^2^ = 2.21 *P* = 0.33	χ^2^ = 1.89 *P* = 0.38	χ^2^ = 1.34 *P* = 0.51	χ^2^ = 2.56 *P* = 0.27	χ^2^ = 1.85 *P* = 0.39

*CHBMS* Champion’s Health Belief Model Scale, *BSE* breast self examination,  Z = Mann–Whitney U test, χ^2^ = Kruskal–Wallis test, *p < 0.05

There was a statistically significant difference between the women’s knowledge of breast cancer and the sub-dimensions of health motivation, BSE barriers, and BSE self-efficacy (*p* = 0.037, *p* = 0.009, *p* = 0.013). For women who have heard of BSE before; CHBMS total score averages (117.0 ± 24.02), health motivation (22.54 ± 4.47), BSE benefits (13.74 ± 4,0.53), and BSE self-efficacy (27.15 ± 8.84) sub-dimensions score averages were found to be significant and high (*p* = 0.007, *p* = 0.001). When women’s previous BSE was compared with the sub-dimensions of CHBMS; a statistically significant difference was found between the sub-dimensions of sensitivity, BSE benefits, BSE, and self-efficacy (*p* = 0.020, *p* = 0.008, *p* = 0.001). There was a statistically significant difference between knowing how to do BSE and the mean scores of sensitivities, BSE benefits, and BSE self-efficacy sub-dimensions (*p* = 0.019, *p* = 0.021, *p* =  0.001). When women’s knowledge of early diagnosis of breast cancer and their scale mean scores were compared; A statistically significant difference was found between BSE benefits and BSE self-efficacy sub-dimensions (*p* =  0.029, *p* =  0.001). BSE benefits (13.62 ± 4.97) and BSE self-efficacy (27.92 ± 9.78) sub-dimensions of the women who knew the early diagnosis of breast cancer were higher than those who did not know. When clinical breast examination status and CHBMS scale mean scores were compared; it was determined that the mean score of women who had clinical breast examination (118.70 ± 29.03) was high and significant (*P* = 0.025) (Table 5).

**Table 5:** Comparison of Young women’s knowledge BC, BSE and BSE practices and CHBMS (n = 413)

Variable	CHBMS Total scores		Sensitivity	Severity	HealthMotivationn	BSE benefits	BSE barriers	BSE self-efficacy
	n (n = 413)	X ± SD	X ± SD	X ± SD	X ± SD	X ± SD	X ± SD	X ± SD
Knowledge of BC								
Yes	146	109.98 ± 28.07	5.59 ± 2.72	18.09 ± 6.64	22.70 ± 8.15	12.96 ± 5.10	24.39 ± 8.13	26.22 ± 9.54
No	267	109.03 ± 25.95	5.78 ± 2.63	19.12 ± 6.36	21.25 ± 7.75	12.61 ± 4.41	26.52 ± 8.41	23.73 ± 8.52
Statistics	Z = 0.81 *P* = 0.416		Z = 0.964 *P* = 0.335	Z = 1.422 *P* = 0.155	Z = 2.081 *P* = 0.037*	Z = 1.192 *P* = 0.233	Z = 2.621 *P* = 0.009*	Z = 2.489 *P* = 0.013*
Have you heard about BSE								
Yes	156	117.0 ± 24.02	5.56 ± 2.58	19.18 ± 6.30	22.54 ± 4.47	13.74 ± 4.53	25.82 ± 7.91	27.15 ± 8.84
No	257	109.54 ± 27.85	5.81 ± 2.70	18.50 ± 6.57	21.29 ± 8.15	12.12 ± 4.64	25.73 ± 8.65	23.07 ± 8.70
Statistics	Z = 2.683 *P* = 0.007*		Z = 0.792 *P* = 0.428	Z = 0.584 *P* = 0.559	Z = 1.485 *P* = 0.001*	Z = 3.619 *P* = 0.001*	Z = 0.118 *P* = 0.906	Z = 4.508 *P* = 0.001*
Knowledge of BSE								
Yes	104	114.42 ± 27.61	5.53 ± 2.60	18.80 ± 6.52	22.88 ± 7.98	13.39 ± 4.76	25.66 ± 8.18	28.13 ± 9.80
No	309	107.66 ± 26.20	5.77 ± 2.68	18.74 ± 6.47	21.38 ± 7.87	12.51 ± 4.61	25.80 ± 8.44	23.42 ± 8.35
Statistics	Z = 2.429 *P* = 0.015*		Z = 0.839 *P* = 0.401	Z = 0.080 *P* = 0.936	Z = 1.860 *P* = 0.063	Z = 1.912 *P* = 0.056	Z = 0.060 *P* = 0.952	Z = 1.860 *P* = 0.063
Have you done BSE before								
Yes	71	114.30 ± 26.35	5.04 ± 2.27	19.25 ± 6.70	22.64 ± 8.25	13.88 ± 4.84	24.84 ± 8.16	28.63 ± 9.95
No	342	108.34 ± 26.68	5.85 ± 2.71	18.66 ± 6.43	21.58 ± 7.85	12.50 ± 4.59	25.96 ± 8.41	23.77 ± 8.52
Statistics	Z = 1.855 *P* = 0.064		Z = 2.321 *P* = 0.020*	Z = 0.799 *P* = 0.424	Z = 1.355 *P* = 0.175	Z = 2.647 *P* = 0.008*	Z = 1.159 *P* = 0.247	Z = 3.645 *P* = 0.001*
To know how to do BSE								
Yes	66	112.57 ± 29.32	4.98 ± 2.23	18.24 ± 6.58	22.27 ± 8.04	13.71 ± 5.10	24.06 ± 8.14	29.30 ± 10.18
No	347	108.75 ± 26.16	5.85 ± 2.71	18.86 ± 6.46	21.66 ± 7.90	12.55 ± 4.56	26.09 ± 8.38	23.72 ± 8.43
Statistics	Z = 1.574 *P* = 0.115		Z = 2.344 *P* = 0.019*	Z = 0.735 *P* = 0.462	Z = 0.837 *P* = 0.403	Z = 2.317 *P* = 0.021*	Z = 1.936 *P* = 0.053	Z = 4.363 *P* = 0.001*
Have you had breast ultrasonography before								
Yes	35	111.28 ± 30.85	6.17 ± 2.78	19.0 ± 6.88	19.97 ± 7.45	13.31 ± 4.90	25.80 ± 7.86	27.02 ± 9.93
No	378	109.19 ± 26.31	5.67 ± 2.65	18.08 ± 6.44	21.93 ± 7.95	12.68 ± 4.64	25.76 ± 8.42	24.38 ± 8.85
Statistics	Z = 0.748 *P* = 0.454		Z = 1.075 *P* = 0.282	Z = 0.601 *P* = 0.548	Z = 1.602 *P* = 0.109	Z = 0.853 *P* = 0.394	Z = 0.150 *P* = 0.880	Z = 1.456 *P* = 0.145
Knowledge about early detection methods of BC								
Yes	75	112.58 ± 28.36	5.52 ± 2.80	18.56 ± 6.09	21.93 ± 8.13	13.62 ± 4.97	25.02 ± 8.83	27.92 ± 9.78
No	338	108.65 ± 26.30	5.76 ± 2.63	18.80 ± 6.56	21.72 ± 7.88	12.54 ± 4.57	25.93 ± 8.27	23.87 ± 8.61
Statistics	Z = 1.699 *P* = 0.089		Z = 1.026 *P* = 0.305	Z = 0.462 *P* = 0.644	Z = 0.461 *P* = 0.645	Z = 2.179 *P* = 0.029*	Z = 0.966 *P* = 0.334	Z = 3.486 *P* = 0.001*
Have you ever had a clinical breast exam								
Yes	24	118.70 ± 29.03	5.87 ± 2.52	19.12 ± 6.75	23.04 ± 8.34	14.41 ± 4.88	27.83 ± 9.58	28.41 ± 10.03
No	389	108.79 ± 26.47	5.70 ± 2.67	18.74 ± 6.46	21.68 ± 7.90	12.63 ± 4.63	25.64 ± 8.28	24.37 ± 8.85
Statistics	Z = 2.242 *P* = 0.025*		Z = 0.501 *P* = 0.616	Z = 0.238 *P* = 0.812	Z = 0.892 *P* = 0.373	Z = 1.928 *P* = 0.054	Z = 1.084 *P* = 0.278	Z = 1.930 *P* = 0.054

*CHBMS* Champion’s Health Belief Model Scale, *BSE* breast self examination,  Z = Mann–Whitney U test, χ^2^ = Kruskal–Wallis test, *p < 0.05

## Discussion

Breast cancer is the first most and common gynecologic cancer among Somali women [33]. When breast cancer is detected early, patients live longer, require less extensive treatment, and the death rate decreases [34]. Therefore, early diagnosis programs are important and BSE is one of the best methods for detecting breast problems [35]. Somalia where health-related resources are limited, and there are never cancer centers, or cancer control programs, so it is important to teach women 20 and older to do BSE [36]. This study aimed to explore which BC and BSE knowledge, beliefs, and practices of young women in Somalia Mogadishu.

The findings of the present study show that Somalian women have poor knowledge of BC. In the studies conducted with women in Saudi Arabia and Ethiopia, similar results were obtained with this study, showing that women’s knowledge of BC is not sufficient [14, 18]. Mamdouh et al. (2014) indicated that Egyptian women also had poor knowledge of BC [37]. Our finding is consistent with previous studies’ results. These results suggest that women in developing countries need education about breast cancer.

Although BSE is a simple method that does not require any invasive intervention, or any tools and can be performed in a short time, and is inexpensive, studies have shown that women do not perform breast examinations at a high rate [8, 38, 39]. It was determined in this study that more than half of the participants had never heard of BSE. Negussie et al. (2015) shows that only %16.5 of women, who are from Ethiopia, heard about BSE [40]. Contrary to these studies, the study conducted in Kenya showed that almost all of the young women had heard of Bse [41]. These results may suggest that demographic differences contribute to the inconsistent findings regarding the age and education level of the participants.

Women’s BSE knowledge was found to be poor in this study. 74.8% of women did not know BSE. Many studies conducted in regions such as Asia, Turkey, Africa, and the Arab peninsula, applications have achieved similar results to our study findings and have shown a very low knowledge of BSE (average 20%) [8, 14, 39, 42–44]. However, in a study conducted with female university students in Ethiopia, the rate of having good BSE knowledge was found to be 49.9% [45]. These results suggest that BSE is a less well-known practice and requires the broad participation of women to improve this situation. In addition, low living standards, lack of health awareness, and less attention to BSE due to socioeconomic disparities in access to health education may contribute to their misunderstanding of BC risk factors and BSE.

In this study, only 17.2% of women performed BSE. Similar to our study result, in a study conducted in India, the rate of female students’ BSE practice was found to be 17% [46]. In line with this result, 31.7% of women in Iraq [47]. 34.9% of women in Ethiopia performed BSE [48]. However, Kratzke et al. conducted a study with women of college age in the United States, obtained slightly better results in contrast to these studies, and stated that the rate of BSE application was 55% [49]. These results consider that low-performance rates can be associated with regional and cultural differences, and women of college age feel healthy and therefore do not need to perform BSE.

According to HBM, women who sense themselves to be susceptible to BC (perceived susceptibility) and who also think that BC is a serious disease (perceived seriousness) are most likely to practice regular BSE. The effect of health beliefs on breast cancer screening of women is important and has been analyzed with this aspect in many studies [20, 50]. The socio-demographic characteristics of individuals can affect their health-related behaviors and attitudes [14]. When sociodemographic characteristics and CHBMS sub-dimensions were compared, our finding showed that a significant difference was found between the sensitivity perception and health motivation sub-dimensions of CHBMS with an economic income. Kirag and Kızılkaya stated in their studies that there is a relationship between economic income and BSE benefits, barriers, and self-efficacy sub-dimensions [51]. These results suggest that economic income is not only effective in receiving healthcare services but also in applying health screenings. In our study, it was determined that there was a significant relationship between marital status and the perception of benefits sub-dimensions of CHBMS. However, in another study, they stated that there was a significant difference in the perception of sensitivity as well as the perception of benefits sub-dimensions of CHBMS [52]. According to these results, married participants think that BSE can be beneficial for early diagnosis. However, in the present study, age, working, and education status was not significant with sub-dimensions of CHBMS. This was also the situation for having children and the number of children.

The literature states that women’s knowledge of BC has a favorable impact on BSE practice. Most women apply to health institutions in the advanced stages of the disease because of their insufficient knowledge about BC and their unawareness of screening methods [14]. In this study, a significant difference was found between women’s knowledge about BC and the CHBMS sub-dimensions of health motivation, barriers, and perception of self-efficacy. In the present study, participants who know BC have high health motivation and confidence perception, and low barrier perception. Erbil and Bölükbaş found that scores of benefits, barriers, confidence, and health motivation sub-dimensions of women who knew BC were statistically significant. In another study conducted in Turkey, unlike these studies, they stated that the participants’ knowledge about BC was only related to their health motivation subscale. These results support the literature and suggest that women’s BC knowledge affects women’s health beliefs, increasing their self-confidence and health motivation in doing BSE.

In our study, a significant difference was found between the participants’ status of hearing BSE before and the subscales of health motivation, BSE benefits, self-efficacy, and total means of scoring CHBMS. In the study conducted with female teachers in Ethiopia, the rate of hearing about BSE was found to be only 16.5%, but no comparison was made with the total means of score CHBMS [40]. These findings suggest that hearing BSE raises awareness about BSE in women and affects health beliefs in doing BSE.

In our study, it was determined that the mean total scores of CHBMS of the participants who knew BSE were high, and a significant difference was found between them. Furthermore, there was no difference between the CHBMS sub-dimensions mean scores of the participants and their knowledge about BSE. Fry and Dunn (2006) showed that the total scores of CHBMS of women who knew BSE were higher than for women who had not acknowledged it [53]. This result is similar to this study. Contrary to these studies, Erbil and Bölükbaş found a significant relationship between women’s knowledge about BSE and the benefits, barriers, confidence, and health motivation sub-dimensions of CHBMS [54].

HBM, there is a positive correlation between practice BSE and health motivation, sensitivity, seriousness, and benefits perceptions, and negative correlations with barriers perceptions [55]. In this study, perceived severity, health motivation, and barriers were not significantly related to BSE practice. However, increased perceived sensitivity, benefits, and self-efficacy were significantly associated with them. Similarly, some studies have shown that women’s higher levels of perceived sensitivity are associated with higher BSE performance [14, 26]. Gözum and Aydın [26] found in their study that perceived benefits as an important determining factor for Turkish women in performing BSE. In contrast, Foxall et al. [56] didn’t find a relation between BSE practice and perceived susceptibility or benefits. These findings propose that women should apply BSE if they are susceptible to breast cancer and might have information about the severity of the disease. In this study, the fact that the participants had insufficient knowledge about BSE and that those who practiced BSE were negligible may suggest that they had low perceptions of sensitivity and seriousness, and high perceptions of BSE barriers. There is a need to increase the sensitivity perceptions and decrease the barriers perceptions of Somali women and to determine the factors affecting their sensitivity through different studies.

Knowing how to do BSE and performing it correctly is important for the effectiveness of the examination. In addition, knowing how to do it affects the situation of performing BSE [14]. Only 16% of the women participating in our study know how to do BSE. The sensitivity, BSE benefits, and BSE self-efficacy of women who knew how BSE was performed were found to be significant and high. The fact that women know how to do BSE affects BSE benefits perception and BSE self-efficacy. Abolfotouh et all. In their study with Saudi women, they determined that among the noticed causes for not performing BSE was not knowing how to examine their breasts or not trusting that they could do it [14]. It is thought that it will be effective to explain how breast examination will be done in the BSE training to be given, by showing it on the practical or training materials. Because knowing how to do BSE is an important condition as it will increase the number of women who do it. At the same time, it is thought that knowing how to do BSE will increase women’s confidence and self-efficacy in their health beliefs.

It is very important for women to know about the early diagnosis and treatment of BC and to have information regarding the hazards and advantages of early diagnosis in terms of BC prognosis. Knowing the early diagnosis has a positive effect on the implementation of BSE. At the same time, it has been determined that women’s knowledge of early diagnosis of BC affects women’s health beliefs, attitudes, and behaviors positively [14, 54]. In our study, although the number of women who knew about the early diagnosis of breast cancer was quite low, it was seen that those who knew had a positive effect on BSE benefits perception and BSE self-efficacy. It is thought that providing breast health programs for women by health professionals and including early diagnosis and diagnosis of breast cancer in these programs will help women gain good health behaviors and maintain their health. It is thought that there is a lack of health education, especially in these societies for breast cancer, which is common in African women, and that health programs should be increased.

WHO has declared that women should have CBE once a year, but more frequently in risky situations [57] In our study, the number of women who had CBE was very low. However, it was determined that the mean CHBMS score of women who had clinical breast examination was high and significant, no significant difference was found with the scale sub-dimensions. Like our study, Darvishpour et all. ın their study with Iranian women, no significant difference was found between the scale sub-dimensions of women who had clinical breast examinations. It was determined that none of the health belief model subscales were effective in increasing CBE performance. [58]. However, in the study of Hajian-Tilaki and Auladi with Iranian women, the perception of benefits, trust, and health motivation sub-dimensions of those who had CBE were found to be significant. They determined that the perception of benefits, confidence, e, and high health motivation affect BCEperformancee [51]. In the study of Kırağı and Kızılkaya with Turkish academic women, it was determined that female academician women who had clinical breast examinations had higher self-efficacy than women who did not [28]. It is thought that women’s clinical breast examination affects their health beliefs.

## Limitations

There were some limitations in this study. First of all, it was a cross-sectional study conducted only with women admitted to a hospital in Mogadishu, Somalia. Therefore, it was not representative of the entire female population in Mogadishu and the results cannot be generalized. The other limitation is that data collection is based on a self-administered survey, so the data may be subject to information bias (recall bias) also another big limitation was the lack concerning articles in addition to publication in journals in Somalia and developing countries; so available references were not used.

## Conclusion

The participants in this study had insufficient knowledge about BC and BSE examination, and BSE practices were very low. Participants did not know how to examine their breasts. Women with a higher perception of BSE sensitivity, benefits, and trust have a lower risk of not performing BSE. These findings show that Somali women need to increase their knowledge, attitude, confidence, and behavior toward BC and BSE. It is important to establish specialized resource centers in Mogadishu in different hospitals and regions to integrate BSE training programs for all women into the health system. Periodic follow-up of women in the community is very important for early diagnosis of BC. Early diagnosis and treatment units in Mogadishu should be established to make women aware of BC and the screening methods. These units should adopt BSE training programs as one of the routine services offered to all women. Healthcare professionals should advise women to be aware of BC, what changes may indicate cancer and how to obtain appropriate resources.

## Data Availability

The datasets generated and analyzed during the current study are not publicly available due to different languages but are available from the corresponding author upon reasonable request.
